# Community versus academic hospital community-acquired pneumonia patients: a nested cohort study

**DOI:** 10.1186/s41479-024-00143-x

**Published:** 2024-11-25

**Authors:** Jennifer L.Y. Tsang, Kian Rego, Alexandra Binnie, Terry Lee, Anne Mccarthy, Juthaporn Cowan, Patrick Archambault, Francois Lellouche, Alexis F. Turgeon, Jennifer Yoon, Francois Lamontagne, Allison Mcgeer, Josh Douglas, Peter Daley, Robert Fowler, David M. Maslove, Brent W. Winston, Todd C. Lee, Karen C. Tran, Matthew P. Cheng, Donald C. Vinh, John H. Boyd, Keith R. Walley, Joel Singer, John C. Marshall, Gregory Haljan, Fagun Jain, James A. Russell, For CAPTIVATE Investigators, Jennifer L.Y. Tsang, Terry Lee, Anne Mccarthy, Juthaporn Cowan, Patrick Archambault, Francois Lellouche, Alexis F. Turgeon, Jennifer Yoon, Francois Lamontagne, Allison Mcgeer, Josh Douglas, Peter Daley, Robert Fowler, David M. Maslove, Brent W. Winston, Todd C. Lee, Karen C. Tran, Matthew P. Cheng, Donald C. Vinh, John H. Boyd, Keith R. Walley, Joel Singer, John C. Marshall, Gregory Haljan, Fagun Jain, James A. Russell

**Affiliations:** 1 Niagara Health Knowledge Institute, Niagara Health, St Catharines, ON Canada; 2 Department of Medicine, McMaster University, Hamilton, ON Canada; 3 Faculty of Applied Health Sciences, Brock University, St Catharines, ON Canada; 4 Critical Care Department, William Osler Health System, Brampton, ON Canada; 5 Algarve Biomedical Centre, Faro, Portugal; 6 Centro Hospitalar Universitário do Algarve, Faro, Portugal; 7 Centre for Advancing Health Outcomes, St. Paul’s Hospital, University of British Columbia, Vancouver, BC Canada; 8 Ottawa Research Institute, University of Ottawa, Ottawa, ON Canada; 9 Centre de recherche intégrée pour un système apprenant en santé et services sociaux, Centre intégré de santé et services sociaux de Chaudière-Appalaches, Levis, Québec Canada; 10 Faculty of Medicine, Université Laval, Québec, Québec Canada; 11 VITAM - Centre de recherche en santé durable, Québec, Québec Canada; 12 Department of Family Medicine and Emergency Medicine, Université Laval, Québec, Québec Canada; 13 Département de médecine, Centre de Recherche de l’Institut Universitaire de Cardiologie et de Pneumologie de Québec, Université Laval, Québec City, QC Canada; 14 Division of Critical Care Medicine, Faculty of Medicine, CHU de Québec-Université Laval Research Center, Population Health and Optimal Health Practices Unit, Trauma- Emergency- Critical Care Medicine, and Department of Anesthesiology and Critical Care Medicine, Université Laval, Québec City, QC Canada; 15 Humber River Hospital, Toronto, ON Canada; 16 University of Sherbrooke, Sherbrooke, QC Canada; 17 Mount Sinai Hospital, University of Toronto, Toronto, ON Canada; 18 Lion’s Gate Hospital, North Vancouver, BC Canada; 19 Memorial University of Newfoundland, St. John’s, NL Canada; 20 Sunnybrook Health Sciences Centre, Toronto, ON Canada; 21 Department of Critical Care, Kingston General Hospital and Queen’s University, Kingston, ON Canada; 22 Departments of Critical Care Medicine, Medicine and Biochemistry and Molecular Biology, Foothills Medical Centre, University of Calgary, Calgary, AB Canada; 23 Division of Infectious Diseases, Department of Medicine, McGill University Health Centre, Montreal, QC Canada; 24 Division of General Internal Medicine, Vancouver General Hospital, Vancouver, BC Canada; 25 Centre for Heart Lung Innovation, St. Paul’s Hospital, University of British Columbia, Vancouver, BC Canada; 26 Division of Critical Care Medicine, St. Paul’s Hospital, University of British Columbia, Vancouver, BC Canada; 27 Department of Surgery, St. Michael’s Hospital, Toronto, ON Canada; 28 Department of Medicine, Surrey Memorial Hospital, Surrey, BC Canada; 29 Black Tusk Research Group, Vancouver, BC Canada

**Keywords:** Community-acquired pneumonia, Mortality, Corticosteroids, Community hospital

## Abstract

**Background:**

Most Canadians receive their care in community hospitals, yet most clinical research is conducted in academic hospitals. This study aims to compare patients with community acquired pneumonia (CAP) treated in academic and community hospitals with respect to their demographics, clinical characteristics, treatments and outcomes.

**Methods:**

This nested observational cohort substudy of the Community Acquired Pneumonia: Toward InnoVAtive Treatment (CAPTIVATE) trial included 1,329 hospitalized adults with CAP recruited between March 1st, 2018 and September 31st, 2023 from 15 Canadian hospitals. Unadjusted and adjusted analyses for age, sex and co-morbidities using logistic, Cox and censored quantile regressions were conducted.

**Results:**

Patients in community hospitals were older (mean [SD] 75.0 [15.7] years vs. 68.3 [16.2] years; *p* < 0.001), were more likely to be female (49.7% vs. 41.0%, *p* = 0.002), and had more comorbidities (75.9% vs. 64.8%, *p* < 0.001). More patients in community hospitals received corticosteroids (49.2% vs. 37.4%, *p* < 0.001). Community hospital patients had a higher likelihood of developing acute respiratory distress syndrome (OR 3.13, 95% CI: 1.87, 5.24, *p* = < 0.001), and acute cardiac injury (OR 2.53, 95% CI: 1.33, 4.83, *p* = 0.005). In unadjusted and adjusted analyses, 28-day mortality difference did not meet statistical significance (OR 1.43, 95% CI: 0.98, 20.7, *p* = 0.062 and OR 1.23, 95% CI: 0.81, 1.87, *p* = 0.332, respective).

**Conclusion:**

Patients with CAP in Canadian community and academic hospitals differed with respect to their age, clinical characteristics, treatments and outcomes, emphasizing the importance of including more community hospitals in clinical research studies to ensure the generalizability of results.

**Supplementary Information:**

The online version contains supplementary material available at 10.1186/s41479-024-00143-x.

## Background

Community hospitals represent over 90% of hospitals in Canada [1]. Although they provide the majority of inpatient clinical care, they do not frequently participate in clinical research studies [2]. Relative to academic hospitals, community hospitals are more likely to be located in suburban and rural communities [3, 4] and are more likely to serve populations with higher proportions of recent immigrants [5] lower socioeconomic status [6–8] and reduced access to subspecialized care [9, 10]. In addition, patients in community hospital tend to be older, with more comorbidities, increased frailty and a higher risk of in-hospital mortality [8]. Thus, research conducted exclusively in academic hospitals may not accurately reflect the patient population in community hospitals.

Community-acquired pneumonia (CAP) affects 330,000 Canadians per year, causing 6,000 deaths and disproportionately affecting older individuals and those with comorbidities [11, 12]. Given the differences in baseline populations between academic and community hospitals, we hypothesized that there are clinically relevant differences in patient baseline characteristics, treatments, and outcomes of patients with CAP in community and academic hospitals.

## Methods

This is a retrospective observational study nested within the Community Acquired Pneumonia: Toward InnoVAtive Treatment (CAPTIVATE) Research program – a multi-centre, pan-Canadian cohort study. Inclusion criteria were hospitalized patients > 18 years of age with an admitting diagnosis of acute CAP defined by having one of fever, chills, leukocytosis, leukopenia; one of cough, sputum, dyspnea; and new infiltrates on chest x-ray consistent with CAP [13–16]. Exclusion criteria were Emergency Department visits without hospital admission, readmissions, and admissions for other reasons. In this nested observational study, we included patients enrolled between March 1, 2018 and September 31, 2023 in 15 Canadian hospitals. We calculated the SMART-COP (systolic blood pressure, multilobar infiltrates, albumin, respiratory rate, tachycardia, confusion, oxygen, and pH) CAP severity score in all patients to understand severity of CAP between community and academic hospitals [17].

The primary outcome was 28-day mortality; patients discharged before day 28 and lost to follow up were assumed 28-day survivors [18]. Secondary outcomes were hospital mortality, Intensive Care Unit (ICU) admission rates, organ dysfunction, and ICU and hospital length of stay. Organ dysfunction was scored first, as frequency of invasive ventilation, vasopressors and Renal Replacement Therapy (RRT) and second, as days alive and free (DAF) of these therapies within the first 14 days [19] determined by subtracting numbers of days on ventilation, vasopressors or RRT from 14. Deaths within 14 days were assigned 0 DAF.

Hospital sites were included by invitation and based on agreement to participate in the study. Hospital status was determined according to the Canadian Institute for Health Information (CIHI) classification, which differentiates hospitals by teaching status [1]. For the purpose of this study, we defined CIHI “teaching” hospitals as “academic” and CIHI “non-teaching” hospitals as “community”. Study outcomes included patient demographics, clinical characteristics, treatments, and clinical outcomes (organ dysfunction, length of stay and mortality).

### Statistical analysis

Baseline and clinical characteristics were compared using Chi-square test, Fisher’s exact test, Analysis of Variance (ANOVA) or Kruskal–Wallis test as appropriate. Unadjusted and adjusted regression analyses (adjusting for pre-defined adjustment factors: age, sex, co-morbidities and CAP severity as measured by modified SMART-COP), logistic, Cox and censored quantile regression were used to compare binary outcomes, survival time and length of stay, respectively [20]. For length of stay analysis, in-hospital deaths were considered as never discharged and censored at the longest observed length of stay [21]. The observed days alive and free (DAF) of ventilation, vasopressors, and renal replacement therapy over the first 14 days post hospital admission data exhibited a U-shape distribution, with most data concentrated at 0 and 14. We thus used 0–1 inflated beta regression to model this data [14]. Results were expressed as odds ratio (OR), hazard ratio (HR), difference in median length of stay and mean difference in DAF with 95% confidence interval (CI).

Approximately 5% of patients had missing data and were therefore excluded from the adjusted regression analyses. Analyses were conducted using SAS 9.4 (SAS Institute Inc., Cary, NC) and R 4.0.4 (R Foundation for Statistical Computing, Vienna, Austria). *P* < 0.05 was considered statistically significant.

### Ethical considerations

This study was approved by Providence Health Care and the University of British Columbia (UBC) Human Research Committee and by each of the participating sites. Collection of anonymized clinical data and discarded plasma from clinical blood tests were deemed low risk and the requirement for informed consent was waived by all the participating REBs.

## Results

### Hospital site characteristics

The CAPTIVATE Research Program included 15 hospital sites across Canada of which 10 (66.7%) were academic hospitals and 5 (33.3%) were community hospitals. Amongst 1,329 patients, 744 (56.0%) were admitted to academic hospitals and 585 (44.0%) to community hospitals, translating to 88 and 142 patients per 1,000 hospital beds respectively. Site characteristics are found in Additional File 1. Median enrollment was numerically higher per site in community sites compared to academic sites (99 [Range: 22–195] vs. 20 [Range: 1-221], *p* = 0.27).

### Patient demographics

Patients enrolled in community hospitals were older (mean [SD] 75.0 [15.7] years vs. 68.3 [16.2] years; *p* < 0.001), more likely to be female (49.7% vs. 41.0%, *p* = 0.002), and were more likely to have comorbidities including chronic cardiac disease, chronic kidney disease, hypertension and diabetes (75.9% vs. 64.8%, *p* < 0.001) (Table 1) than patients enrolled in academic hospitals. Specifically, community hospital patients had higher proportions of chronic kidney disease (23.1% vs. 15.3%, *p* < 0.001), hypertension (60.8% vs. 48.4%, *p* < 0.001), chronic neurological disorders (16.1% vs. 9.2%, *p* < 0.001), rheumatologic disorders (19.1% vs. 12.5%, *p* < 0.001) and dementia (14.0% vs. 8.0%, *p* < 0.001) (Table 1). Conversely, academic hospital patients had a higher proportion of acquired immune deficiency syndrome and human immunodeficiency virus infection (AIDS/HIV) (1.5% vs. 0.2%, *p* = 0.011) (Table 1).

**Table 1:** Patient demographics and clinical characteristics

Variable	Community (*n* = 585)	Academic (*n* = 744)	*P* value
Province, n/total (%)			< 0.001
AB	0/585 (0.0)	1/744 (0.1)	
BC	109/585 (18.6)	188/744 (25.3)	
NF	0/585 (0.0)	6/744 (0.8)	
ON	281/585 (48.0)	259/744 (34.8)	
QC	195/585 (33.3)	290/744 (39.0)	
Sex, n/total (%)			0.002
Unknown	1/585 (0.1)	3/744 (0.4)	
Male	294/585 (50.3)	437/744 (59.0)	
Female	290/585 (49.7)	304/744 (41.0)	
Age, years			< 0.001
Mean (SD)	75.0 (15.7)	68.3 (16.2)	
Median (IQR)	77.0 (66.0, 87.0)	70.0 (60.0, 80.0)	
Range	(20.0, 103.0)	(20.0, 103.0)	
Co-morbidities, n/total (%)			
Any of the four^a^	443/584 (75.9)	481/742 (64.8)	< 0.001
Chronic cardiac disease	239/585 (40.9)	275/742 (37.1)	0.159
Chronic kidney disease	135/584 (23.1)	113/740 (15.3)	< 0.001
Hypertension	355/584 (60.8)	359/741 (48.4)	< 0.001
Diabetes	156/585 (26.7)	183/741 (24.7)	0.414
Chronic pulmonary disease (not asthma)	172/585 (29.4)	243/740 (32.8)	0.181
Asthma^b^	56/585 (9.6)	78/741 (10.5)	0.567
Liver disease	26/585 (4.4)	32/739 (4.3)	0.920
Chronic neurological disorder	94/584 (16.1)	68/739 (9.2)	< 0.001
Malignant neoplasm	125/585 (21.4)	135/740 (18.2)	0.155
Chronic hematologic disease	30/585 (5.1)	48/740 (6.5)	0.297
AIDS / HIV	1/585 (0.2)	11/732 (1.5)	0.011
Obesity (as defined by clinical staff)	33/585 (5.6)	44/734 (6.0)	0.786
Rheumatologic disorder	112/585 (19.1)	92/738 (12.5)	< 0.001
Dementia	82/585 (14.0)	59/738 (8.0)	< 0.001
Malnutrition	10/582 (1.7)	8/727 (1.1)	0.340
Positive culture^c^, n/total (%)			
* Streptococcus pneumoniae*	131/574 (22.8)	152/728 (20.9)	0.399
* Staphylococcus aureus*	28/573 (4.9)	43/723 (5.9)	0.405
* Haemophilus influenza*	15/572 (2.6)	25/725 (3.4)	0.393
* Klebsiella/Enterobacter*	6/573 (1.0)	12/724 (1.7)	0.351
Other	8/573 (1.4)	8/726 (1.1)	0.633
Influenza, n/total (%)	80/574 (13.9)	87/727 (12.0)	0.291
Admitted to ICU on hospital admission day, n/total (%)	41/584 (7.0)	63/743 (8.5)	0.326
Organ support on admission day, n/total (%)			
Oxygen therapy	287/583 (49.2)	324/732 (44.3)	0.073
Invasive mechanical ventilation	30/585 (5.1)	45/744 (6.0)	0.471
Renal replacement therapy	4/583 (0.7)	4/742 (0.5)	0.737
Vasopressors	35/585 (6.0)	45/744 (6.0)	0.960
Modified SMART-COP Score^d^			0.233
Unknown	3	46	
0	352 (60.5)	465 (66.6)	
1	129 (22.2)	124 (17.8)	
2	67 (11.5)	73 (10.5)	
3	21 (3.6)	22 (3.2)	
≥ 4	13 (2.2)	14 (2.0)	

^a^Chronic cardiac disease, chronic kidney disease, hypertension or diabetes

^b^Diagnosed by a physician

^c^Blood or sputum, within 48 h before or after hospital admission

^d^Modified SMART-COP = Systolic blood pressure, respiratory rate, tachycardia, oxygen. Chest x-ray, albumin, Glasgow Coma Score, PaO2/FiO2 and arterial pH were not included as the score component as they were not consistently captured in the database

### Clinical characteristics

Rates of laboratory-confirmed bacterial and influenza CAP were comparable between patients enrolled in community and academic hospitals (Table 1). The frequency of organ support and need for oxygen therapy at admission were also similar, as was the rate of ICU admission. There was no difference in Modified SMART-COP scores [17].

### Hospital interventions and treatments

Almost all patients received antibiotics in both settings (99.7% vs. 97.8%, *p* = 0.005). The proportions of patients receiving any corticosteroids (49.2% vs. 37.4%, *p* < 0.001) were higher in community hospitals relative to academic hospitals (Table 2), however the times to initiation were similar (Table 2). The proportion of patients receiving organ support was similar between community and academic hospitals.

**Table 2:** Hospital interventions

Intervention	Community (*n* = 585)	Academic (*n* = 744)	*P* value
Co-intervention while hospitalized, n/total (%)			
Antiviral agent	66/583 (11.3)	85/741 (11.5)	0.932
Remdesivir	7/583 (1.2)	16/741 (2.2)	0.185
Antibiotic	583/585 (99.7)	728/744 (97.8)	0.005
Corticosteroid	288/585 (49.2)	278/744 (37.4)	< 0.001
Dexamethasone	46/585 (7.9)	32/744 (4.3)	0.006
Antifungal agent	24/583 (4.1)	29/744 (3.9)	0.840
Time to initiation of corticosteroid, days, n/total (%)			0.434
Unknown	21/288 (7.3)	8/278 (2.9)	
0	138/288 (47.9)	154/278 (55.4)	
1	65/288 (22.6)	61/278 (21.9)	
> 1	64/288 (22.2)	55/278 (19.8)	
Organ support while hospitalized, n/total (%)			
Invasive mechanical ventilation	57/585 (9.7)	74/744 (9.9)	0.902
Renal replacement therapy	16/583 (2.7)	15/742 (2.0)	0.388
Vasopressors	64/585 (10.9)	77/744 (10.3)	0.729

### Clinical outcomes

**Fig. 1:**
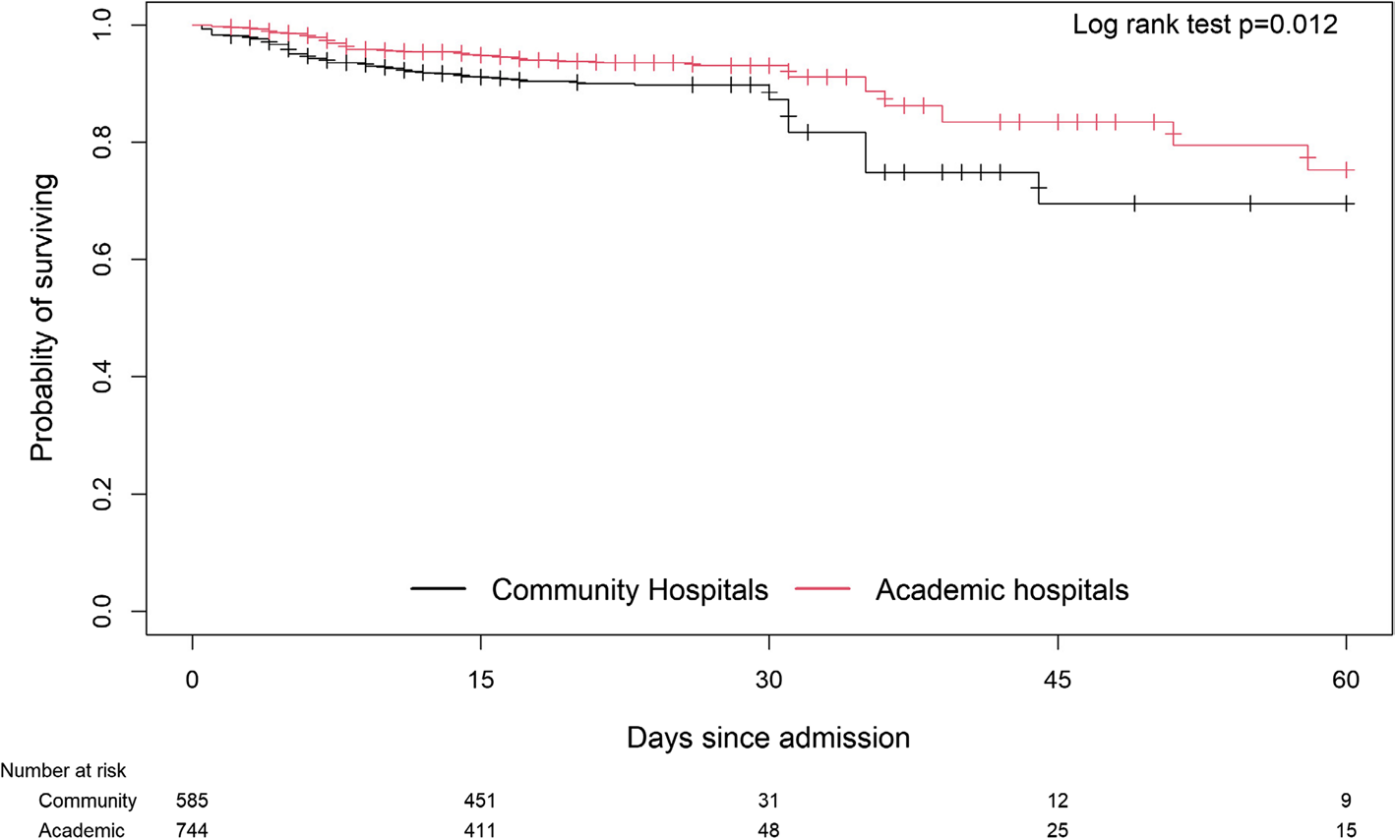
Kaplan-Meier survival estimates of hospitalized CAP patients in community versus academic hospitals

In unadjusted analyses, community hospital patients had higher in-hospital mortality (OR 1.91, 95% CI: 1.28, 2.85, *p* = 0.001) than academic hospital patients (Tables 3 and 4). Kaplan-Meier survival curves also showed significantly better survival for academic hospital patients (Log Rank *p* = 0.012, Fig. 1). However, when analyses were adjusted for age, sex, co-morbidities and CAP severity, the difference in survival was no longer significant (Table 4).

**Table 3:** Clinical outcomes for community hospital patients vs academic hospital patients

Variable	Community (*n*=585)	Academic (*n*=744)	*P *value
Mortality, n/total (%)			
Primary: 28-day	63/585 (10.8)	58/744 (7.8)	0.061
In-hospital	63/585 (10.8)	44/744 (5.9)	0.001
Admitted to ICU, n/total (%)	90/585 (15.4)	121/744 (16.3)	0.663
During the first 14 days, DAF^a^ of, mean (SD)			
Invasive mechanical ventilation	12.4 (4.2)	13.0 (3.3)	0.037
Renal replacement therapy	12.7 (4.0)	13.3 (2.9)	<0.001
Vasopressors	12.5 (4.0)	13.3 (3.1)	0.018
Hospital length of stay – deceased (time to death)			0.084
n/total (%)	63/585 (10.8)	44/744 (6.0)	
Median, days (IQR)	6.0 (4.0, 14.0)	8.0 (6.0, 18.0)	
Hospital length of stay – survivors			0.092
n/total (%)	522/585 (89.2)	700/744 (94)	
Median, days (IQR)	6.5 (4.0, 11.0)	6.0 (4.0, 11.0)	
ICU length of stay – deceased^b^			0.208
n/total (%)	18/585 (3.1)	17/744 (2.3)	
Median, days (IQR)	11.5 (4.0, 21.0)	7.0 (4.0, 8.0)	
ICU length of stay – survivors^b^			0.012
n/total (%)	72/585 (12.3)	93/744 (12.5)	
Median, days (IQR)	7.5 (4.0, 12.0)	5.0 (3.0, 10.0)	
Septic shock, n/total (%)	41/581 (7.1)	48/734 (6.5)	0.711
Acute respiratory distress syndrome, n/total (%)	53/584 (9.1)	25/741 (3.4)	<0.001
Acute kidney injury, n/total (%)	118/579 (20.4)	105/733 (14.3)	0.004
Acute cardiac injury, n/total (%)	29/585 (5.0)	19/711 (2.7)	0.030

^a^DAF = Days Alive and Free

^b^Among those who were admitted to ICU

**Table 4:** Comparison of outcomes for community hospital patients vs academic hospital patients by regression analysis

	Unadjusted analysis		Adjusted analysis	
Outcome	Odds/hazard ratio (95% CI)	*P* value	Odds/hazard ratio (95% CI)	*P* value
Primary outcome: 28-day mortality	1.43 (0.98, 2.07)	0.062	1.23 (0.81, 1.87)	0.332
In-hospital death	1.91 (1.28, 2.85)	0.001	1.38 (0.90, 2.11)	0.142
Time to death	1.63 (1.11, 2.40)	0.013	1.20 (0.79, 1.82)	0.389
Admitted to ICU^a^	0.94 (0.70, 1.26)	0.669	1.17 (0.83, 1.64)	0.367
Organ support while hospitalized				
Invasive mechanical ventilation	0.98 (0.68, 1.41)	0.909	1.28 (0.86, 1.92)	0.226
RRT	1.36 (0.68, 2.75)	0.386	1.68 (0.76, 3.71)	0.197
Vasopressors	1.07 (0.75, 1.51)	0.723	1.19 (0.81, 1.75)	0.387
Organ support during first 14 days				
Invasive mechanical ventilation	1.01 (0.70, 1.45)	0.978	1.33 (0.88, 2.00)	0.176
RRT	1.38 (0.65, 2.92)	0.402	1.77 (0.76, 4.10)	0.182
Vasopressors	1.06 (0.74, 1.51)	0.752	1.18 (0.80, 1.75)	0.398
Septic shock	1.09 (0.71, 1.67)	0.704	1.25 (0.78, 2.01)	0.346
Acute respiratory distress syndrome	2.83 (1.74, 4.59)	<0.001	3.13 (1.87, 5.24)	<0.001
Acute kidney injury	1.53 (1.15, 2.04)	0.004	1.23 (0.89, 1.70)	0.199
Acute cardiac injury	1.88 (1.05, 3.37)	0.034	2.53 (1.33, 4.83)	0.005
	Unadjusted analysis		Adjusted analysis	
Outcome	Difference in median/mean (95% CI)	*P* value	Difference in median/mean (95% CI)	*P *value
Hospital length of stay	1.0 (0.2, 1.9)	0.021	0.5 (-0.2, 1.3)	0.173
ICU length of stay	3.0 (0.3, 5.7)	0.031	1.7 (-1.9, 5.3)	0.363
DAF^a^ first 14 days				
Invasive mechanical ventilation	-0.6 (-1.0, -0.2)	0.003	-0.5 (-1.0, -0.1)	0.020
RRT^a^ or dialysis	-0.7 (-1.1, -0.3)	<0.001	-^b^	
Vasopressors	-0.6 (-1.0, -0.2)	0.003	-0.4 (-0.8, 0.0)	0.056

^a^DAF = days alive and free, ICU = intensive care unit, RRT = renal replacement therapy

^b^Adjusted regression analysis was not feasible numerically as few patients received renal replacement therapy during the first 14 days

Community hospital patients had greater frequencies of acute respiratory distress syndrome (ARDS) (9.1% vs. 3.4% *p* < 0.001), acute kidney injury (AKI) (20.4% vs. 14.3%, *p* = 0.004), and acute cardiac injury (ACI) (5.0% vs. 2.7%, *p* = 0.030). The differences for ARDS and ACI remained statistically significant after regression adjustment (Table 4).

Community hospital patients had fewer Days Alive and Free (DAF) [19] of invasive mechanical ventilation, vasopressors and renal replacement therapy during the first 14 days of hospitalization in unadjusted analysis. While overall hospital length of stay was similar between community and academic hospital patients, ICU survivors had a greater length of ICU stay in community hospitals (7.5 vs. 5.0 days, *p* = 0.012).

## Discussion

In our multicenter cohort study, there were important baseline differences between patients from community versus academic hospitals. We observed that patients admitted to Canadian community hospitals with CAP were older, more often female, and had more co-morbidities than their academic hospital counterparts. They also had higher severity of illness and a higher proportion of patients developed ARDS, AKI and ACI. In unadjusted analyses, in-hospital mortality was higher in community hospital patients. However, logistic regression analyses revealed that differences at baseline accounted for the higher mortality. With respect to treatments, almost all patients in both settings received antibiotics, while community hospital patients were more likely to receive corticosteroids. However, they were equally likely to receive organ support with mechanical ventilation, vasopressors, and renal replacement therapy. Notably, nearly 95% of patients in both settings had a Modified SMART-COP score of 0–2, reflecting the similar rate of ICU admissions, vasopressor support and mortality (after adjusted analyses) between both groups. ICU stay was longer for community ICU survivors than for academic ICU survivors.

Community hospital patients with CAP often have higher severity of illness compared to their academic hospital counterparts [8, 22, 23]. In unadjusted analyses, in-hospital mortality was higher amongst community hospital patients but after adjustment for age, sex, comorbidities and CAP severity, this gap was no longer detected; suggesting that differences in baseline patient characteristics and severity of illness account for much of the observed mortality difference. Data on social determinants of health, including income, education, and race, were not collected in CAPTIVATE but may also have contributed to poorer outcomes among community hospital patients [24–26]. With respect to medical treatments, community hospital patients were more likely to receive corticosteroids than their academic hospital counterparts which may reflect differences in baseline patient characteristics and also practice patterns that could impact outcomes. Overall, these findings demonstrate that the patient populations in community and academic hospitals differ with respect to their baseline and clinical characteristics, suggesting the need to include more community hospital patients in clinical research to ensure the generalizability of results to the wider population.

Although community hospitals represent more than 90% of Canadian hospitals, they represented only 33% of the hospitals included in this study. The underrepresentation of community hospitals is commonly observed in research studies, including clinical trials [2]. Yet, research results generated in academic hospitals are routinely used to guide care in community hospitals. Our results show that in patients with CAP, baseline characteristics, the provision of treatment and clinical outcomes differ between community and academic hospitals. Considering these differences, it is vital to increase access to research for patients in community hospitals in order to generate clinical evidence that is more applicable to their care. Insufficient research infrastructure, inadequate funding, a lack of research experience and limited organizational commitment to research are known barriers to community hospital research participation [27–32]. However, a recent study demonstrated that community hospitals participating in a randomized control trial had similar consent rates, enrolment rates and protocol adherence to academic hospitals [8]. Moreover, in this study, we observed less missing data in community hospitals compared to academic hospitals. Thus, community hospitals have the ability to participate in clinical trials with similar trial metrics as well as strong potential for study recruitment.

The strengths of this study include the large sample size and the substantial representation of community hospital patients. Additionally, the waived consent model reduced the likelihood of bias in patient recruitment. Limitations included the post-hoc retrospective study design, the relatively small number of community hospitals that participated and that our data represents Ontario, BC and Quebec with very small representation from Alberta and Newfoundland. Although community hospital site participation was low, community hospital patients represented almost half of the patients in the study which suggests that these observed differences may be generalizable among CAP patients in community versus academic hospitals. However, it should be noted that the community hospitals included in this study may not be entirely representative of the characteristics (i.e., size, participation in research) of all Canadian community hospitals. While the focus of the current study was to observe rather than explain differences in patient characteristics, an additional limitation is that data on social determinants of health and race/ethnicity, which may have impacted patient outcomes, were not collected. Furthermore, the definition of “teaching” and “non-teaching” hospital may not be precise, noting that some community hospitals have trainees.

## Conclusions

In conclusion, community hospital patients with CAP enrolled in the CAPTIVATE trial differed from academic hospital patients with respect to their baseline and clinical characteristics, treatments and outcomes. After adjusted analyses, in-hospital mortality was the same between community and academic hospital patients however, community hospital patients were older and presented with more comorbidities. These results emphasize the need to increase community hospital participation in studies focused on the causes and treatment of pneumonia. Moreover, these findings call into question the generalizability of clinical research results that are generated from studies conducted exclusively in academic hospitals, highlighting the need to increase community hospital patient representation in clinical research. Increasing community hospital participation in health research has the potential to improve study recruitment, accelerate study completion, and improve the generalizability of study results for more Canadians.

## Supplementary Information

Supplementary Material 1.

## Data Availability

The datasets used and/or analysed during the current study are available from the corresponding author on reasonable request.

## Abbreviations

CAP: Community Acquired Pneumonia
ICU: Intensive Care Unit
CAPTIVATE: Community Acquired Pneumonia: Toward InnoVAtive Treatment
SMART-COP: Systolic blood pressure, Multilobar infiltrates, Albumin, Respiratory rate, Tachycardia, Confusion, oxygen, and PH
DAF: Days Alive and Free
ANOVA: Analysis of Variance
OR: Odds Ratio
HR: Hazard Ratio
CI: Confidence Interval
AIDS/HIV: Acquired Immune Deficiency Syndrome and Human Immunodeficiency Virus
ARDS: Acute Respiratory Distress Syndrome
AKI: Acute Kidney Injury
ACI: Acute Cardiac Injury
