# Supplementary material for: Community versus academic hospital community-acquired pneumonia patients: a nested cohort study
Source: Pneumonia (Nathan). 2024 Nov 25;16:31. doi: 10.1186/s41479-024-00143-x (PMC11587589; doi:10.1186/s41479-024-00143-x)
Supplement: Supplementary file 1 — Supplementary Material 1. [file 41479_2024_143_MOESM1_ESM.docx]

**Additional File 1**

**Supplementary Table 1. Hospital Characteristics**

| Hospital name | Province | Hospital Classification^*^ | Number of beds^*^ | | |
| --- | --- | --- | --- | --- | --- |
|  |  |  | Total^†^ | ICU^†^ | Other Acute^‡^ |
| Foothills Medical Centre | AB | Academic | 1001 | 62 | 771 |
| St. Paul’s Hospital | BC | Academic | 425 | 35 | 301 |
| Surrey Memorial Hospital^§^ | BC | Academic | 624 | 41 | 423 |
| Lions Gate Hospital | BC | Community | 544 | 9 | 179 |
| Health Sciences Centre - St. John’s | NL | Academic | 331 | 31 | 247 |
| Humber River Hospital | ON | Community | 685 | 57 | 524 |
| Mount Sinai Hospital (Sinai Health) | ON | Academic | 744 | 24 | 195 |
| The Ottawa Hospital | ON | Academic | 1134 | 70 | 853 |
| Niagara Health System | ON | Community | 884 | 56 | 491 |
| Kingston Health Sciences Centre | ON | Academic | 539 | 78 | 377 |
| Sunnybrook Health Sciences Centre | ON | Academic | 1109 | 106 | 508 |
| CHU de Québec- Université Laval | QC | Academic | 1204 | 126 | 899 |
| CIUSSS de l'Estrie-Centre hospitalier universitaire de Sherbrooke | QC | Academic | 1166 | 86 | 807 |
| Centre universitaire de santé McGill | QC | Academic | 828 | 125 | 552 |
| CISSS-CA - Hôtel-Dieu de Lévis^\|\|^ | QC | Community | **____** | **____** | **____** |

^*^CIHI classifies hospitals into teaching and non-teaching. For the purpose of this manuscript, we label teaching hospitals as “academic hospitals” and non-teaching hospitals as “community” hospitals. Hospital classification and number of beds retrieved from Canadian Institute for Health Information (CIHI) (2023).

^†^ Number of beds does not include neonatal intensive care unit (NICU), contracted-out in-patient long-term care and surgical services, or residential care units (CIHI, 2023).

^‡^ Includes acute-care in-patient beds for services including: nursing inpatient administration and medical resources, medical and surgical nursing units, operating room and post-anesthetic recovery rooms, and palliative nursing units (CIHI, 2023)

^§^ CIHI (2023) data classifies Surrey Memorial Hospital as a teaching site however, for the purpose of the current study, this site is categorized as a community hospital because it is not a research-oriented institution

^||^Information on number of beds not provided by CIHI dataset (CIHI, 2023).
